# The supportive care needs and its influencing factors among thyroid cancer patients after surgery: A cross-sectional study

**DOI:** 10.3389/fsurg.2023.1108425

**Published:** 2023-03-08

**Authors:** Ming Cai, Juxiang Gou

**Affiliations:** West China School of Nursing, Sichuan University/Thyroid Surgery Department, West China Hospital, Sichuan University, Chengdu, China

**Keywords:** SCNS, supportive care needs, TC, thyroid cancer, influencing factors, function, surgery

## Abstract

**Objective:**

To study the supportive care needs (SCNS) of thyroid cancer (TC) patients after surgery, and to identity its influencing factors.

**Methods:**

By convenience sampling method, total of 115 patients undergoing thyroid surgery who met the inclusion criteria from May 2021 to July 2021 were selected as the research objects. The general information questionnaire, Supportive Care Need Survey Short-Form (SCNS-SF34), functional subscale of European Organization for Research and Treatment of Cancer Quality of Life Questionnaire-Core 30 (EORTC QLQ-C30) were used to investigate these patients.

**Results:**

The average score for the items of SCNS-SF34 in 102 TC patients was 2.15 ± 0.71. The domain with the highest item average score was “health system information needs” (2.48 ± 1.17). The domain with the highest unsatisfied rate was “psychological needs” (69.6%). The item with the highest average score was “fear of cancer spreading” in the psychological needs domain (2.80 ± 1.48). The scores of physical function (*r* = −0.431, *P* < 0.001), role function (*r* = −0.428, *P* < 0.001), cognitive function (*r* = −0.531, *P* < 0.001), emotional function (*r* = −0.388, *P* < 0.001), social function (*r* = −0.464, *P* < 0.001) were correlated with the total score of SCNS-SF34. The univariate analysis of SCNS-SF34 scores showed that TC patients who were women (*t* = 2.013, *P *= 0.047), older (F = 1.353, *P *= 0.013), and with longer hospital stays (F = 3.705, *P *= 0.028) had higher demand of SCNS. The results of multiple linear regression analysis showed that the significant variables that entered the regression equation were gender, age, length of stay in hospital, cognitive function and social function (*P *< 0.05).

**Conclusion:**

TC patients after surgery have many SCNS in different domains. It is necessary to focus on women, older patients, patients with long hospital stays and poor functioning. The implementation of a supportive care screening tool is recommended and the individualized interventions need to be developed to provide targeted support and care.

## Introduction

In recent years, the number of patients with thyroid cancer (TC) has exploded, making it the solid malignant tumor with the fastest-growing incidence (1). In 2020, TC is the most commonly diagnosed malignancy of the endocrine system and the ninth leading cancer, and accounted for 586,000 new cases worldwide (2). Surgery is the main method of treating TC (3). Postoperative patients will have different needs in many aspects such as physical, psychological, spiritual and social (4–6). Unmet needs can increase negative emotions such as anxiety and depression, reduce diagnosis and treatment compliance and disease coping ability, thereby seriously affecting physical and mental health and quality of life (7–9). Supportive Care Needs (SCNS) refers to the general term for all kinds of help that cancer patients and their families may need to prevent, control or alleviate various complications and side effects in addition to medical, surgical and other interventions (10). The essence of Supportive Care is to comprehensively assess the needs of cancer patients, proactively provide targeted support and care, and maximize the satisfaction of patients’ needs, thereby improving their quality of life. In order to ensure that health professionals are meeting the holistic care needs of cancer patients and prioritize resource allocation to those aspects of care that need improvement, it is essential to identify what those needs are and evaluate whether care delivery meets those expectations. To the best of our knowledge, there are few studies focused specifically on SCNS in TC patients. The researches on the needs for TC patients were mainly conducted in Europe and the United States (11, 12), and there were problems such as inconsistent use of validated instruments and study designs which leaded to different results. The nature and degree of patients’ SCNS are influenced by their cultures (13). This cross-sectional study was the first conduced in mainland China to investigate the SCNS and related influencing factors of TC patients after surgery to provide the basis for formulating targeted care and support measures.

## Materials and methods

### Setting and participants

From May 2021 to July 2021, the convenience sampling method was used to select TC patients newly admitted to the Department of Thyroid Surgery, West China Hospital, Sichuan University for surgery as the research subjects. The inclusion criteria were (1) patients aged between 18 and 80 years; (2) patients with TC who had chosen surgical treatment; (3) patients were able to understand the survey content and express their inner feelings clearly; (4) patients had given informed consent to the research content, voluntarily participated and could actively cooperate. The exclusion criteria were: (1) patients accompanied by other malignancies; (2) patients had other serious illness. The eliminative criteria were: (1) patients who did not complete surgery; (2) patients dropped out of the survey. The sample size of this study is based on the sample size estimation method of multiple regression analysis (14). The sample size is at least 10 times the number of main independent variables. Considering the 15% loss rate of the sample, the sample size = 10 × 10 × (1 + 15%) = 115 cases.

### Measures

The general information questionnaire included basic demographics: gender, age, occupation, marital status, length of hospital staying, surgical method. The Supportive Care Need Survey Short-Form (SCNS-SF34) (15) was used to assess the SCNS of patients. This questionnaire can comprehensively assess the needs of all aspects of cancer patients. Au et al. (16) translated SCNS-SF34 into Chinese and Cronbach alpha reliability coefficients exceeded 0.7 for all domains. Five domains of the SCNS-SF34 include physical and daily living, psychological, sexual, care and support, and health system information. Each item adopts Likert 5-level scoring method, 1 point for no demand, 2 points for there is demand and the current demand has been met, 3, 4, and 5 points indicate that the degree of demand is low, medium, and high, respectively. The higher the score, the higher the level of demand. Domain average score = d total score/number of patients; item average score = domain total score/patient number/number of items in this domain; unmet need rate = number of patients with unmet needs (score ≥3 points)/patient case number. The functional subscale of European Organization for Research and Treatment of Cancer Quality of Life Questionnaire-Core 30 (EORTC QLQ-C30) (17, 18) was used to assess the functioning of patients. This questionnaire has been widely used to measure the quality of life of Chinese cancer patients, with a Cronbach alpha reliability coefficient of 0.810. In this study, the functional subscale was selected for evaluation. The functional subscale has a total of 15 items and 5 functional domains (physical, role, cognitive, emotional and social). All items have 4 response categories (not at all, a little, quite a bit and very much), directly score 1–4 points. Add the scores of the items included in each domain and divide by the number of included items to get the score in the field. After linear transformation, all subscales range from 0 to 100. A higher score means better functioning. Participants were asked to to fill out two questionnaires based on their current situation. The overall Cronbach's alpha SCNS-SF34 and EORTC QLQ-C30 were 0.943 and 0.853, which indicates a reliable instrument in the study.

### Data collection

The whole process of the investigation was completed by the investigator himself, and the patients were included on the day of admission. The purpose, method and significance of the study were introduced to the patients, and all patients signed an informed consent form before the investigation. The general data of the patients were investigated and collected before discharge. SCNS-SF34 and EORTC QLQ-C30 functional subscale questionnaires were conducted on the second day after the operation face-to-face. Each survey included an information letter and instructions to complete the questionnaires. The investigator was available to answer further questions if requested. Questionnaires were distributed and collected on the spot. All data were entered after double-checking.

### Statistical analysis

Statistical analysis was performed using SPSS 20.0 (SPSS Inc., Chicago, IL, USA). Internal consistency of the modified tool was assessed using Cronbach's alpha. K-S test was used to verify whether the data conformed to normal distribution. The qualitative data with a normal distribution were expressed as mean ± standard deviation. The quantitative data were expressed as frequency and percentage. The qualitative data with a normal distribution and homogeneity of variance were compared using the independent-sample t test between two groups, and ANOVA test between multiple groups. Pearson correlation analysis was used to analysis the correlation between the scores of SCNS-SF34 and EORTC QLQ-C30. Multiple linear regression analysis was carried out with the factors affecting the SCNS-SF34 score as independent variables. *P* < 0.05 was considered statistically significant.

### Ethical considerations

Ethical approval to conduct the study was granted from the Ethics Committee on Biomedical Research, West China Hospital of Sichuan University [Ethics Approval Number 2020(947)].

## Results

A total of 115 cases were included in this study, of which 9 were discharged without surgery and 4 were excluded from the survey, resulting in a final sample of 102 cases. All patients completed the questionnaires. Among the 102 patients, the pathological type of 100 patients were papillary thyroid carcinoma, the pathological type of 1 was poorly differentiated thyroid carcinoma and the pathological type of 1 was medullary thyroid carcinoma, and there were no patients who had undergone reoperation. In this study, most of the participants were female (73.5%), under the age of 40 (67.7%), married (79.4%) and employee (59.8%). Demographic and disease characteristics are presented in Table 3.

The average score for the items of SCNS-SF34 was 2.15 ± 0.71. The scores of each domain of SCNS-SF34 are shown in Tables 1, 2. The domain with the highest average score was “health system information needs”, the domain with the highest unmet rate was “psychological needs”. The results of the average score of items were ranked to determine the ten most prevalent SCNS requiring help across all domains (Table 2). The item with the highest score was “fears about the cancer spreading” in the psychological needs domain.

**Table 1:** The score of participants in each domain of SCNS-SF34 (*n* = 102).

	Domain average score (mean ± SD)	Item average score (mean ± SD)	Unmet need rate (%)
Physiological and daily	9.59 ± 3.82	1.91 ± 0.76	54.9
Psychological	21.09 ± 9.41	2.15 ± 0.94	69.6
Sexual	4.33 ± 2.18	1.44 ± 0.73	19.6
Care and support	10.77 ± 4.76	2.11 ± 0.95	48.0
Health system information	27.25 ± 12.82	2.48 ± 1.17	55.9

SD=, standard deviation.

**Table 2:** Top 10 items with the highest score of SCNS-SF34 (*n* = 102).

Priority	Issue	Domain	Score (mean ± SD)
1	Fears about the cancer spreading	P	2.80 ± 1.48
2	Being informed about things you can do to help yourself to get well	H & I	2.69 ± 1.41
3	Having one member of hospital staff with whom you can talk about all aspects of your condition, treatment and follow-up	H & I	2.66 ± 1.44
4	Being informed about your test results as soon as feasible	H & I	2.61 ± 1.35
5	Being treated in a hospital or clinic that is as physically pleasant as possible	H & I	2.57 ± 1.37
6	Being informed about cancer which is under control or diminishing (that is, remission)	H & I	2.54 ± 1.43
7	Being adequately informed about the benefits and side-effects of treatments before you choose to have them	H & I	2.50 ± 1.32
8	Worrying about your loved ones worrying about you	P	2.47 ± 1.20
9	Being given explanation of those tests for which you would like explanations	H & I	2.44 ± 1.23
10	Being treated like a person not just another case	H & I	2.42 ± 1.32

*P*, psychological; H & I, health system and information; SD, standard deviation.

Univariate analysis of SCNS-SF34 is shown in Table 3. There was significant difference in score of patients with different gender, age and length of hospital stay (*P *< 0.05). The scores of the physical function, role function, cognitive function, emotional function, and social function were negatively correlated with the score of SCNS-SF34 (*P* < 0.05), as shown in Table 4. Taking the SCNS-SF34 total score as the dependent variable, the variables with statistically significant differences in the univariate analysis and correlation analysis in this study were used as independent variables for multiple linear regression analysis. The variable assignments were as follows: gender (male = 0, female = 1), age (≤40 = 0, 41–50 = 1, ≥51 = 2), length of hospital stay (≤5 = 0, 6–7 = 1, ≥8 = 2). The results showed that the significant variables entering the regression equation were gender, age, length of hospital stay, cognitive function and social function (*P *< 0.05), which together explained 43.3% of the variance in the equation, as shown in Table 5.

**Table 3:** Univariate analysis of SCNS-SF34 (*n* = 102).

Variables	*n* (%)	Score (mean ± SD)	Statistics	*P*-value
Gender			*t *= 2.013	0.047
Male	27 (26.5)	65.07 ± 21.56		
Female	75 (73.5)	75.89 ± 24.73		
Age(years)			*F *= 1.353	0.013
≤40	69 (67.6)	70.59 ± 23.12		
41–50	22 (21.6)	75.91 ± 26.21		
≥51	11 (10.8)	82.55 ± 27.01		
Marital status			*F *= 0.551	0.578
Unmarried	16 (15.7)	67.94 ± 18.59	* *	
Married	81 (79.4)	74.32 ± 25.46		
Divorced/Separated/Widowed	5 (4.9)	68.40 ± 22.11		
Occupation			*F *= 0.290	0.833
Farmer	5 (4.9)	73.80 ± 28.54		
Employee	61 (59.8)	74.59 ± 23.09		
Freelance	18 (17.6)	68.61 ± 24.10		
Retired/unemployed	18 (17.6)	71.94 ± 28.73		
Length of hospital stay (days)			*F *= 3.705	0.028
≤5	24 (23.5)	61.58 ± 27.16		
6–7	54 (52.9)	76.06 ± 21.12		
≥8	24 (23.5)	79.67 ± 25.45		
Total Thyroidectomy			*t* = −0.630	0.530
Yes	53 (52.0)	74.49 ± 26.79		
No	49 (48.0)	71.45 ± 21.46		
Neck lymph node dissection			*t* = −0.822	0.413
Yes	16 (15.7)	77.63 ± 25.99		
No	86 (84.3)	72.17 ± 24.04		

SD, standard deviation.

**Table 4:** Pearson correlation analysis between scores of SCNS-SF34 and EORTC QLQ-C30 (*n* = 102).

Domain	Score (mean ± SD)	SCNS	
		*r*	*P*-value
Physical function	77.06 ± 18.66	−0.431	<0.001
Role function	79.06 ± 22.33	−0.428	<0.001
Cognitive function	78.84 ± 19.58	−0.531	<0.001
Emotional function	83.01 ± 17.07	−0.388	<0.001
Social function	77.45 ± 22.45	−0.464	<0.001

SD, standard deviation.

**Table 5:** Multiple regression analysis of SCNS (*n* = 102).

	Coefficients	Standard Error	*t* Stat	*P*-value	Lower 95%	Upper 95%
Intercept	116.175	16.107	7.249	<0.001	84.769	148.739
Gender	11.091	4.635	2.393	0.019	1.886	20.296
Age	7.144	2.771	2.578	0.012	1.641	12.648
Length of hospital stay	3.146	2.778	1.133	0.040	2.370	8.662
Cognitive function	−0.515	0.122	−4.222	<0.001	−0.757	−0.273
Social function	−0.353	0.108	−3.281	0.001	−0.566	−0.139

*R*^2^*^ ^*= 0.477, Adjusted *R^2 ^*= 0.433, *F *= 10.622, *P *< 0.001.

## Discussion

The main purpose of our study is to investigate the SCNS of TC patients after surgery, so we choose SCNS-SF34 questionnaires which can comprehensively assess the needs of all aspects of cancer patients. Previous studies have shown that patients’ needs are closely related to their functional level. EORTC QLQ-C30 questionnaires can fully evaluate the function of patients. To identity the influencing factors of SCNS, we choose to apply this function scale to explore whether the functions will affect the needs.

As shown in Table 1, except for the domain of sexual needs, the unmet needs of the other four domains were all over 45%. This means that TC patients have many SCNS in different domains. This study shows that TC patients after surgery had the strongest demand in the domain of “health system information needs” and echoed the results of a previous studie (12). One previous study (19) have shown that have shown that patients need to be informed about postoperative complications, follow-up treatment and rehabilitation guidance after surgery. They also expect to be informed that the cancer has been relieved, and they also want to receive relevant guidance on how to deal with uncomfortable symptoms and return to society. Therefore, in the postoperative stage, health care professionals should identify the types of information needs of patients in a timely manner, take the needs of patients as the starting point, and formulate personalized health education programs that combines offline and online to meet their needs for health information. The highest unmet rate was “psychological needs”, which was the same as the research results of Sakamoto et al. (20). Psychological stress is a common experience of cancer patients. After diagnosis, patients will have various psychological problems such as worrying about the effect of treatment, fear of cancer spread, and guilt towards their loved ones (21). This suggests that health care professionals should pay attention to the psychological care of patients during the perioperative period, understand their psychological state and give corresponding guidance. We should help patients reduce negative emotions through various ways, such as mindfulness-based stress reduction, incentive nursing. The item of the highest score was “fear of cancer spread” and the same results were also found in patients with gynaecological cancer (22), indicating that the psychological burden of patients is further increased due to lack of disease-related knowledge. Therefore, it is necessary to organically combine health education and psychological care in clinical work to improve the psychological state of patients. The lower scores in the three domains of “physiological and daily life needs”, “sexual needs” and “care and support needs” may be related to the fact that most of the patients in this study were papillary thyroid carcinomas, which resulted in a good prognosis (23).

This study showed that the SCNS of women was higher than that of men, the same result was found in other studies (13, 24). Compared with men, women are characterized by rich emotions, sharp minds, and strong family values. They are more susceptible to factors such as disease and surgery, and have higher levels of negative emotions and psychological experiences (25). Therefore, gender need to be considered when formulating care plans for patients, and the multiple needs of female patients need to be fully evaluated and met. The SCNS increased by age. The physiological function of the patients gradually declines as the age increases, and the physical and mental discomfort caused by the operation is more prominent. Therefore, the older patients have more needs in health education, basic nursing and psychological counseling. Health care professionals should pay more attention to the needs of older patients, improve their disease awareness, improve their physical discomfort and reduce their emotional fluctuations through effective interventions. The longer the hospitalization time, the higher the SCNS. The patients with longer hospitalization time are often related to factors such as older age, complicated surgery and poor postoperative recovery (26) and the patients themselves have higher needs in terms of physiology and daily life. At the same time, prolonged hospitalization will increase the psychological and economic burden of patients, which will further increase their needs. Therefore, health care professionals should adjust caring strategies in a timely manner and provide targeted support with the prolongation of hospitalization time. The functional status of patients is negatively correlated with SCNS, especially cognitive function and social function are closely related to SCNS. Similar correlations have been shown in other cancer patients (27, 28). Therefore, in clinical practice, health care professionals need to provide appropriate psychological, physical, and social support to help patients relieve physical symptoms, reduce negative emotions, improve cognitive and social support levels, and achieve the purpose of improving physical and mental functions. It is worth noting that this study showed that the surgical approach is not related to the degree of need, indicating that any type of TC patients should receive the same attention.

This notion of individually assessing patients for their SCNS is supported by a research investigating the patterns and predictors of unmet SCNS in cancer patients (29). They suggest that addressing these deficits is essential to achieving optimal cancer care and satisfaction but in order to do that, there is a need to develop a consistent process to identify the needs of patients using a standardized instrument in clinical practice.

This study had certain limitations. First, it was a single-center study with limited sample representativeness. Second, the study used a cross-sectional survey, and the content of the study was limited. It is expected that more empirical studies will be conducted in the future to further explore the SCNS of TC patients.

## Conclusion

Our study shows that TC patients after surgery have many SCNS in different domains, and it is necessary to focus on female patients, older patients, patients with longer hospital stays and poor functional. The implementation of a comprehensive but succinct supportive care screening tool is recommended for ensuring that SCNS are identified. Health care professionals should formulate personalized caring interventions and provide targeted support according to the different needs of patients. Early identification and management of needs may help to reduce the burden on health system resources.

## Data Availability

The raw data supporting the conclusions of this article will be made available by the authors, without undue reservation.

## References

[1] Fitzmaurice C Abate D Abbasi N Abbastabar H Abd-Allah F Abdel-Rahman O Global, regional, and national cancer incidence, mortality, years of life lost, years lived with disability, and disability-adjusted life-years for 29 cancer groups, 1990 to 2017: a systematic analysis for the global burden of disease study. JAMA Oncol. (2019) 5(12):1749–68. 10.1001/jamaoncol.2019.2996 31560378 PMC6777271

[2] Sung H Ferlay J Siegel RL Laversanne M Soerjomataram I Jemal A Global cancer statistics 2020: GLOBOCAN estimates of incidence and mortality worldwide for 36 cancers in 185 countries. CA Cancer J Clin. (2021) 71(3):209–49. 10.3322/caac.21660 33538338

[3] Zhu J Tian W Xu Z Jiang K Sun H Wang P Expert consensus statement on parathyroid protection in thyroidectomy. Ann Transl Med. (2015) 3(16):230. 10.3978/j.issn.2305-5839.2015.08.20 26539447 PMC4598451

[4] Morley S Goldfarb M. Support needs and survivorship concerns of thyroid cancer patients. Thyroid. (2015) 25(6):649–56. 10.3978/j.issn.2305-5839.2015.08.20 25809716

[5] Ma Y. Supportive needs of postoperative breast cancer patients and its influencing factors. J. Clin. Nurs. (2019) 18(4):11–4. 10.3969/j.issn.1671-8933.2019.04.004

[6] Dhillon VK Silver Karcioglu A Bloom G Randolph G Lango M. What the thyroid cancer patient wants to know: ThyCa survey by the American Head and Neck Society Endocrine Surgery Section. Head Neck. (2020) 42:2496–504. 10.1002/hed.26185 32530116

[7] Husson O Mols F Oranje WA Haak HR Nieuwlaat WA Netea-Maier RT Unmet information needs and impact of cancer in (long-term) thyroid cancer survivors: results of the PROFILES registry. Psychooncology. (2014) 23:946–52. 10.1002/pon.3514 24619907

[8] Beekers N Husson O Mols F van Eenbergen M van de Poll-Franse LV. Symptoms of anxiety and depression are associated with satisfaction with information provision and internet use among 3080 cancer survivors: results of the PROFILES registry. Cancer Nurs. (2015) 38(5):335–42. 10.1097/NCC.0000000000000184 25226516

[9] Shin YW Choi YM Kim HS Kim DJ Jo HJ O'Donnell BF Diminished quality of life and increased brain functional connectivity in patients with hypothyroidism after total thyroidectomy. Thyroid. (2016) 26(5):641–9. 10.1089/thy.2015.0452 26976233 PMC4939446

[10] Fitch MI. Supportive care needs of patients with advanced disease undergoing radiotherapy for symptom control. Can Oncol Nurs J. (2012) 22(2):84–100. 10.5737/1181912x2228491 22764585

[11] Hyun YG Alhashemi A Fazelzad R Goldberg AS Goldstein DP Sawka AM. A systematic review of unmet information and psychosocial support needs of adults diagnosed with thyroid cancer. Thyroid. (2016) 26(9):1239–50. 10.1089/thy.2016.0039 27350421

[12] Dionisi-Vici M Fantoni M Botto R Nervo A Felicetti F Rossetto R Distress, anxiety, depression and unmet needs in thyroid cancer survivors: a longitudinal study. Endocrine. (2021) 74(3):603–10. 10.1007/s12020-021-02786-y 34143334 PMC8571224

[13] Jabbarzadeh Tabrizi F Rahmani A Asghari Jafarabadi M Jasemi M Allahbakhshian A Unmet supportive care needs of Iranian cancer patients and its related factors. J Caring Sci. (2016) 5(4):307–16. 10.15171/jcs.2016.032 28032075 PMC5187551

[14] Ni P Chen J Liu N. The sample size estimation hi quantitative nursing research. Chin J Nurs. (2010) 45(4):378–80. 10.3761/j.issn.0254-1769.2010.04.037

[15] Boyes A Girgis A Lecathelinais C. Brief assessment of adult cancer patients’ perceived needs: development and validation of the 34-item supportive care needs survey(SCNS-SF34). J Eval Clin Pract. (2009) 15(4):602–6. 10.1111/j.1365-2753.2008.01057.x 19522727

[16] Au A Lam WW Kwong A Suen D Tsang J Yeo W Validation of the Chinese version of the short-form supportive care needs survey questionnaire(SCNS-SF34-C). Psychooncology. (2011) 20(12):1292–300. 10.1002/pon.1851 22114044

[17] Aaronson NK Ahmedzai S Bergman B Bullinger M Cull A Duez NJ The European organization for research and treatment of cancer QLQ-C30: a quality-of-life instrument for use in international clinical trials in oncology. J Natl Cancer Inst. (1993) 85(5):365–76. 10.1093/jnci/85.5.365 8433390

[18] Wan C Chen M Zhang C Tang X Meng Q Zhang X. The Chinese version of EORTC QLQ-C3 form in evaluation of quality of life for patients with cancer. J Pract Oncol. (2005) 20(4):353–5. 10.3969/j.issn.1001-1692.2005.04.028

[19] Harrison JD Young JM Price MA Butow PN Solomon MJ. What are the unmet supportive care needs of people with cancer? A systematic review. Support Care Cancer. (2009) 17(8):1117–28. 10.1007/s00520-009-0615-5 19319577

[20] Sakamoto N Takiguchi S Komatsu H Okuyama T Nakaguchi T Kubota Y Supportive care needs and psychological distress and/or quality of life in ambulatory advanced colorectal cancer patients receiving chemotherapy: a cross-sectional study. Jpn J Clin Oncol. (2017) 47(12):1157–61. 10.1093/jjco/hyx152 29077931

[21] Sanson-Fisher R Girgis A Boyes A Bonevski B Burton L Cook P. The unmet supportive care needs of patients with cancer. Supportive care review group. Cancer. (2000) 88(1):226–37. 10.1002/(sici)1097-0142(20000101)88:1<226::aid-cncr30>3.3.co;2-g 10618627

[22] Williams N Griffin G Farrell V Rea A Murray K Hauck YL. The supportive care needs of women experiencing gynaecological cancer: a Western Australian cross-sectional study. BMC Cancer. (2018) 18(1):912. 10.1186/s12885-018-4812-9 30241476 PMC6151067

[23] Ryu J Ryu YM Jung YS Kim SJ Lee YJ Lee EK Extent of thyroidectomy affects vocal and throat functions: a prospective observational study of lobectomy versus total thyroidectomy. Surgery. (2013) 154(3):611–20. 10.1016/j.surg.2013.03.011 23932596

[24] Miniotti M Bassino S Fanchini L Ritorto G Leombruni P. Supportive care needs, quality of life and psychological morbidity of advanced colorectal cancer patients. Eur J Oncol Nurs. (2019) 43:101668. 10.1016/j.ejon.2019.09.009 31593821

[25] Wang X Wang S Jia Y Xu Y Guo Y. Status and influencing factors of spiritual needs of cancer patients: a survey study. J Nurs Sci. (2020) 35(3):74–6. 10.3870/j.issn.1001-4152.2020.03.074

[26] Zhao L Yin S Yang Y Wang L Yang J Zheng S Risk factors associated with prolonged postoperative length of stay of patients with gastric cancer. Chin J Oncol. (2020) 42(2):150–4. 10.3760/cma.j.issn.0253-3766.2020.02.012 32135651

[27] He S Shan C Xu W Chen R. Correlation investigation between supportive care needs and quality of life in patients with lung cancer. Int J Nurs. (2019) 38(17):2715–8. 10.3760/cma.j.issn.1673-4351.2019.17.009

[28] Miniotti M Bassino S Fanchini L Ritorto G Leombruni P. Supportive care needs and the impact of loss of functioning and symptom burden on the quality of life in patients with advanced colorectal cancer. Oncol Res Treat. (2022) 45(5):262–71. 10.1159/000521753 34983050

[29] Okediji PT Salako O Fatiregun OO. Pattern and predictors of unmet supportive care needs in cancer patients. Cureus. (2017) 9(5):e1234. 10.7759/cureus.1234 28620565 PMC5467772

